# Self-Organizing Cyclolinear Organosilicon Polymers in Bulk and on the Surface of Water ^†^

**DOI:** 10.3390/ijms140918215

**Published:** 2013-09-05

**Authors:** Nataliya N. Makarova, Tat’yana V. Astapova, Alexander I. Buzin, Arkady P. Polishchuk, Nataliya V. Chizhova, Irina M. Petrova

**Affiliations:** 1 Nesmeyanov Institute of Organoelement Compounds, Russian Academy of Sciences, 28 Vavilova str., Moscow 119991, Russian Federation; E-Mails: nmakar@ineos.ac.ru (N.N.M.); baastapov@mail.ru (T.V.A.); n.chizhova2010@yandex.ru (N.V.C.); impetr2013@yandex.ru (I.M.P.); 2 Enikolopov Institute of Synthetic Polymer Materials, Russian Academy of Sciences, 70 Profsoyuznaya str., Moscow 117393, Russian Federation; 3 Institute of Physics, National Academy of Science of Ukraine, 46 Nauky av., Kiev 03028, Ukraine; E-Mail: ark.nau@gmail.com

**Keywords:** amphiphilic cyclolinear polymers, mesomorphic polyorganocarbosiloxanes, Langmuir films

## Abstract

Cyclolinear organocarbosiloxane polymers with varying content and location of (CH_2_)_n_ groups in the monomer unit were synthesized by reactions of heterofunctional polycondensation and polyaddition of difunctional organocyclosiloxanes and organocyclocarbosiloxanes. Their bulk properties were studied by differential scanning calorimetry and X-ray structural analysis. It was shown that on introduction of CH_2_ groups into the methylcyclohexasiloxane unit, the polymer retains the ability to self-organize with formation of a mesomorphic state in a wide temperature range, while on introduction of (CH_2_)_2_ fragments in a cyclotetrasiloxane unit or in a bridge connecting two methylcyclotetra(hexa)siloxane units it does not. Comparison of the X-ray data of dihydroxy derivatives of decamethylcyclohexasiloxane and decamethyl-5-carbocyclohexasiloxane with packing of cyclolinear organosilicon polymers in bulk shows that the polymer inherits the layered type of crystalline structure typical for monomers. Langmuir films of cyclolinear polymethylcarbosiloxanes with different design of monomer units were studied as well. It was revealed that all polymers form monomolecular films at the air/water interface, excluding those having longer hydrophobic fragment than hydrophilic ones. The ability to form multilayers depends on the surroundings of Si atom in the bridge between the cycles.

## 1. Introduction

In a previous study, [1] one of the authors obtained cyclolinear (CL) polyorganosiloxanes (POS) with different sizes and conformations of the organocyclosiloxane moiety in the repeating unit and studied how the polymer chain tacticity, organic substituents, and the molecular weight of POS influenced their physicochemical properties [2–5]. It was found that many CL homopolymers and block copolymers in the bulk formed a mesomorphic state existing in the temperature range from a few tens to a few hundreds of degrees [1,4,6]. A study of the ability of CL polymethylsiloxanes (PMS) to spread over the surface of water at the air/water interface revealed that these polymers formed monomolecular films and multilayers [7,8]. Linear polydimethylsiloxane (PDMS) is known to form monomolecular films (Langmuir monolayers) on the surface of water on compression [9,10]. Zisman’s assumptions of the formation of Langmuir layers by PDMS were developed by Noll [11,12] who assumed that all siloxane bonds in PDMS interacted with water with the formation of a monolayer and that methyl groups were directed outward from the water surface. On further compression, the PDMS Langmuir film is rolled into a helical structure and a second step in the surface pressure appears in the π-*A* isotherm. Polydiorganosiloxanes with asymmetrical substituents also form monomolecular films, but their π-*A* isotherms show only one step. Based on the results obtained in the studies of some CL POS with different structures of the repeating unit of homo- and copolymers, it was assumed that the formation of multilayers at the air/water interface upon lateral compression was due to the ability of some CL POS to form a mesomorphic state in the bulk [8]. In contrast to PDMS, transition of CL POS to the mesomorphic state proceeds via the formation of discrete ordered multilayer structures whose thickness is a multiple of the thickness of the monomolecular film.

In studies on the behavior of CL POS on the water surface, the mechanism of layer formation at the air/water interface have been poorly studied. In particular, this concerns the influence of (i) the ratio of the number of SiO bonds to the number of SiC bonds (hereafter, the SiO/SiC ratio) in the main chain and (ii) organic substituents in interaction with the surface of water. The mechanism of H-bond formation by SiO groups of methylcyclosiloxanes with the surface of water still remains unclear, because CL PMS with cyclotetra-, cyclopenta-, and cycloheptasiloxane moieties do not form multilayers [7,8].

To elucidate the effect of the size and conformation of the cyclosiloxane moiety and the structure of the linker in the unit of CL POS (in particular, CL PMS), to distinguish the contribution of each factor to the formation of the mesomorphic state in the bulk and in monomolecular Langmuir films, new CL polyorganocarbosiloxanes (POCS) were synthesized.

## 2. Results and Discussion

### 2.1. Relation between Crystalline Packing of the Monomer and Self-Organization of the Polymer in Bulk

A study of poly[oxy(2,4,4,6,8,8-hexaorganocyclotetrasiloxane-2,6-diyl)]s with different substituents showed that the formation of the mesomorphic state with the 2D type of packing and the existence of the region of this state depended on the intermolecular interactions of the side groups [4]. Table 1 lists the phase transition temperatures of the CL polymers with the structural Type **I** bearing different alkyl substituents. The narrowest existence region of the mesomorphic state was found for the mainly syndiotactic polymer with methyl substituents (syndiotactic **I**-**Me**). An atactic Polymer **I**-**Me** does not form the mesophase. In contrast to the atactic Polymer **I**-**Me**, atactic poly[oxy(2,4,4,6,6,8,10,10,12,12-decamethylcyclohexasiloxane-2,8-diyl)] (**II**-**Me**) forms the mesomorphic phase in a wide temperature interval; the introduction of phenyl substituents leads to an increase in the isotropization temperature *T*_i_ [13]. It should be noted that the tacticity of the polymer chain in the CL POS with the structural Type **II** has little effect on the appearance of the mesophase with the 1D packing type [2,4]. Yet another property of CL POS with the structural Type **II** is noteworthy: Namely, this is the effect of the molecular weight on the change in *T*_i_ [4,5]. Unlike linear polydiorganosiloxanes, the mesomorphic state in CL POS **II** appears at much lower degrees of polymerization (*n* = 3–5) [14–16]. Table 2 presents the phase transition temperatures of the CL POS with the structural Type **II**. Thus, CL POS with symmetric chain units of the structural Types **I** and **II** form the mesomorphic state. Comparing their ability to form the mesophase with that of linear polydiethylsiloxane, one can state that the major contribution to self-organization of the macromolecules of CL polymers comes from the cyclic fragments.

**Figure:**
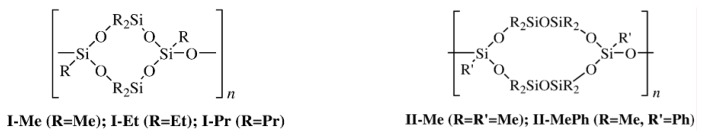


To assess the influence of oxygen atoms in the unit of CL PMS on the ability to self-organize, the bridging oxygen atoms between the cyclosiloxane moieties in **I**-**Me**, **I**-**Et**, and **II**-**Me** were replaced by some other species.

**Scheme 1:**
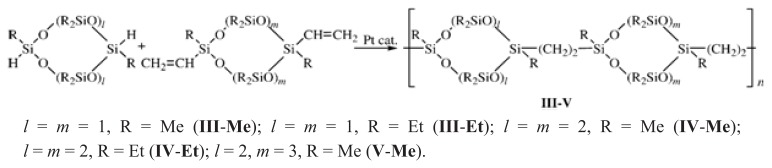
Synthesis of CL PMCS **III**–**V**.

Cyclolinear polyorganocarbosiloxanes with ethylene groups instead of oxygen atoms >SiMeOMeSi< as bridges between rings were synthesized by the polyaddition reaction of dihydridocycloorganotetra(hexa,octa)siloxanes with divinylcycloorganotetra(hexa)siloxanes bearing methyl and ethyl substituents in the presence of platinum complex catalysts (the Karstedt and Speier catalysts, dicyclopentadienylplatinum dichloride) and their reduced forms following Scheme 1 [17].

^1^H and ^13^C nuclear magnetic resonance (NMR) studies revealed that the polyaddition reaction obeys Farmer’s rule and that the degree of formation of CH_2_CH_2_ bridges between the rings is higher than 95% [17]. Using differential scanning calorimetry (DSC) and X-ray analysis, it was found that the CL POCS with the hexaorganocyclotetrasiloxane moiety in the chain unit bearing methyl (**III**-**Me**) and ethyl (**III**-**Et**) substituents were crystalline and amorphous, respectively. Compound **IV**-**Me**, *i.e.*, the CL POCS with the decamethylcyclohexasiloxane fragment of the chain unit was also amorphous in the temperature interval studied.

Tables 1 and 2 list the transition temperatures of the CL POCS with ethylene bridges between the cyclotetra- (**III**-**Me** and **III**-**Et**) and cyclohexasiloxane moieties (**IV**-**Me** and **IV**-**Et**), as well as that of the copolymer with alternating cyclohexa- and cyclooctasiloxane moieties (**V**-**Me**). All samples except **IV**-**Et** exhibit no mesomorphic properties.

**Figure 1:**
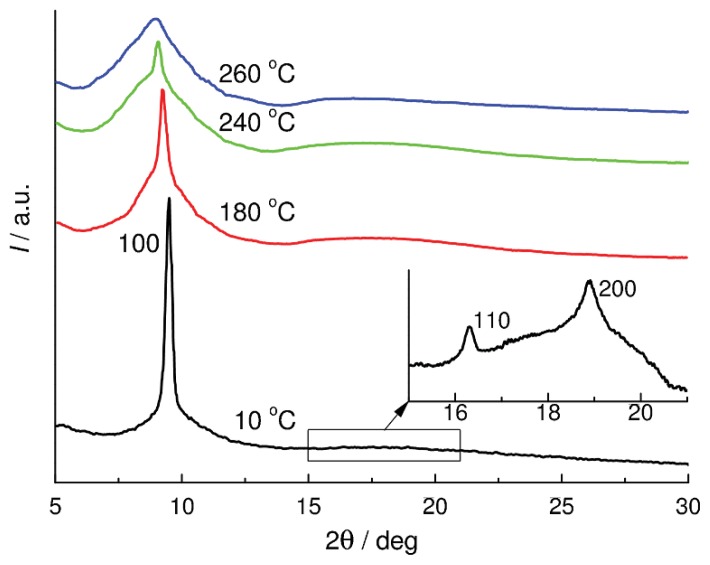
X-ray diffraction patterns of CL POCS **IV**-**Et** at different temperatures.

Thus, the ability of CL POCS with methyl substituents to self-organize is suppressed upon replacement of the oxygen bridge by the CH_2_CH_2_ unit. Attempts to change the ratio of the number of SiO groups to the number of SiCH_2_ groups (hereafter, the SiO/SiCH_2_ ratio) in the main chain by varying the ring size from cyclotetrasiloxane to cyclooctasiloxane did not lead to recovery of the ability to self-organize with the formation of a mesomorphic state. The ability to self-organize in a wide temperature range appears only upon introducing ethyl substituents into the CL POCS with the Structure **IV** [17], similarly to the case for the CL POS **I**-**Et** (Table 1). The close values of *T*_g_, *T*_i_, and the existence regions of the mesomorphic state of the CL POS **I**-**Et** and CL POCS **IV**-**Et** are noteworthy. This means that the contribution of structural changes in the main chain becomes less significant owing to strengthening of intermolecular interactions involving organic substituents. The X-ray diffraction pattern of Polymer **IV**-**Et** is typical of mesomorphic systems. It exhibits a single narrow reflection in the region 2θ_m_ = 9.43° (*d*_1_ = 9.37 Å). At low temperatures, two weak reflections were revealed additionally, sin 2θ_m_:sin 2θ_I_:sin 2θ_II_ = 1:3:4 (Figure 1). The aforesaid suggests that in the mesomorphic state, CL POCS **IV**-**Et** has a 2D packing type with hexagonal symmetry up to *T*_i_. This packing type in the mesophase is confirmed by the high gradient of the *d*_m_(*T*) dependence. The structure-forming element of the mesophase is the macromolecule.

Thus, replacement of the oxygen atom by the (CH_2_)_2_ unit between the cyclotetra- or cyclohexasiloxane moieties in the polymer chain leads to the loss of the ability of CL POCS to self-organize with the formation of 1D packing in the case of CL polymethylcarbosiloxanes (PMCS). Additional factors, such as the introduction of ethyl substituents into CL POS and CL POCS, produce intermolecular interactions favoring the recovery of the ability to form the mesomorphic state. This is accompanied by a decrease in the effect of both cyclosiloxane conformations and the chemical structure of the bridge between the cyclosiloxane moieties on *T*_i_.

**Scheme 2:**
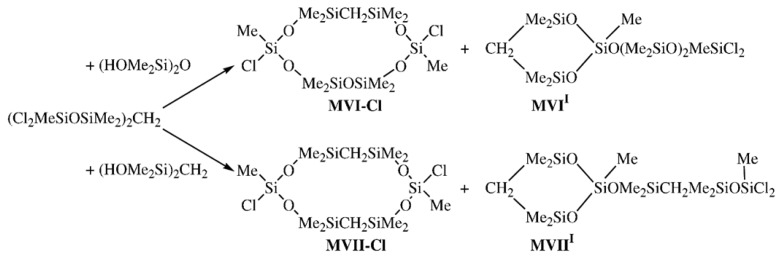
Synthesis of dichloromethylcyclocarbosiloxane structural isomers **MVI**-**Cl**, **MVI^I^** and **MII**-**Cl**, **MVII^I^**.

Single oxygen atoms in decamethylcyclohexasiloxane were replaced by CH_2_ units by the stepwise condensation of 1,3-dihydroxytetramethyldisilylmethylene with methyltrichlorosilane followed by the interaction of 1,1,7,7-tetrachloro-1,3,3,5,5,7-hexamethyl-4-carbodisiloxane with various organosilicon 1,3-dihydroxy derivatives following Scheme 2 [18].

Since our attempt to separate two structural isomers, **MVII**-**Cl** and **MVII^I^**, had failed, hydrolysis of a mixture of these isomers gave a mixture of hydroxy derivatives; 2,8-dihydroxy-2,4,4,6,6,8,10,10,12,12-decamethyl-5,11-dicarbacyclohexasiloxane (**MVII**-**OH**) was isolated by fractional recrystallization. To reveal specific features associated with the conformational changes in the cyclohexasiloxane moiety upon the introduction of one and two methylene units, two model monomeric compounds, *viz.*, 2,8-dihydroxy-2,4,4,6,6,8,10,10,12, 12-decamethyl-5-carbacyclohexasiloxane (**MVI**-**OH**) and 2,8-dihydroxy-2,4,4,6,6,8,10,10,12, 12-decamethyl-5,11-dicarbacyclohexasiloxane (**MVII**-**OH**), were studied [19,20] by X-ray analysis assuming that the unit of CL POCS would inherit the carbocyclosiloxane conformation. In addition, Compounds **MVI**-**OH** and **MVII**-**OH** were used to compare their behaviors on the surface of water under lateral compression in order to evaluate the co-operative contribution of the Me_2_SiOSiMe_2_ and MeSi(OH)O fragments to the surface of water.

**Figure 2:**
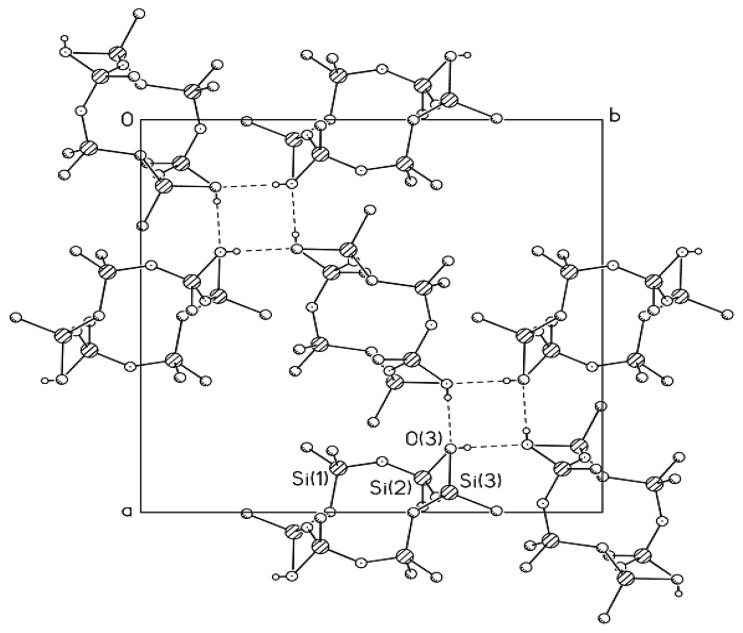
Molecular packing in the crystal of Compound **MII**-**OH** (projection on the *ab* plane). Dashed lines denote intermolecular H-bonds that link molecules to form tetramers.

It was found that replacement of an oxygen atom by a CH_2_ unit in *trans*-2,8-dihydroxy-2,4,4,6,6,8,10,10,12,12-decamethylcyclohexasiloxane (**MII**-**OH**) lead to small conformational changes (Figure 2). The angle ∠ SiCSi = 122.1° in the ring noticeably deviated from the tetrahedral angle [14], whereas the angle ∠ SiCSi in 1,3-disilacyclobutane compounds was close to 90° [21]. Based on the results obtained, one can state with certainty that the angls ∠ SiCSi in the cyclosiloxane systems can vary within a wide range. In the crystal, four-membered rings comprised of H-bonded *trans*-2,8-dihydroxy-2,4,4,6,6,8,10,10,12,12-decamethyl-5-carbacyclohexasiloxane molecules form puckered layers.

**Figure 3:**
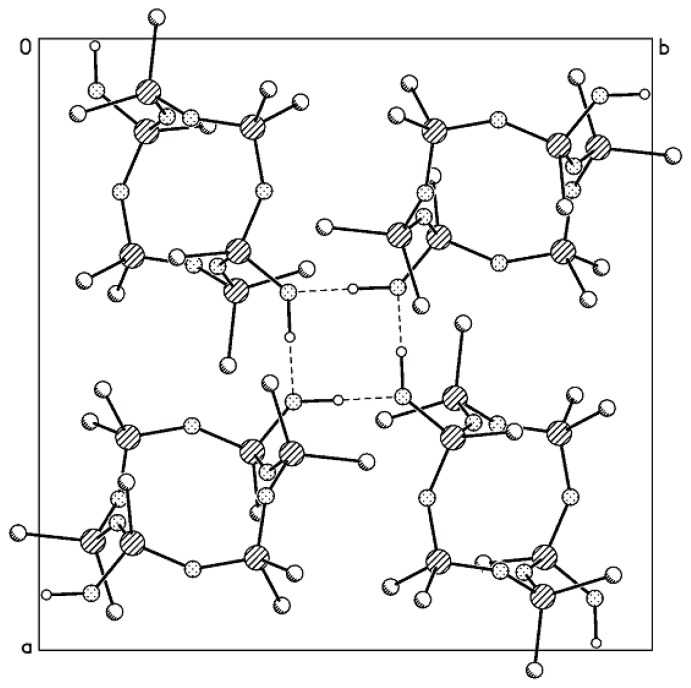
Molecular packing in the crystal of Compound **MVII**-**OH** (projection on the *ab* plane). Dashed lines denote intermolecular H-bonds that link molecules to form tetramers.

Generally, the conformation of Compound **MVII**-**OH** with two CH_2_ units can be represented by a crown, but in most cases these moieties adopt a complex folded conformation which can hardly be described using some conventional pattern. The valence angle ∠ SiCSi = 123.0° significantly differs from the tetrahedral angle, being, however, close to the angle ∠ SiCSi = 122.1° in Compound **MVI**-**OH** with one CH_2_ unit (*cf*. a value of 146.4° for the valence angle ∠ SiOSi in the *trans-*Compound **MII**-**OH** [22]). The packing of molecules in the crystal of *trans*-Compound **MVII**-**OH** (Figure 3) is similar to that in the crystal of **MVI**-**OH**. The molecules are also linked by H-bonds to form tetramers surrounding a fourfold inversion axis. A similar kind of molecular ordering was also found for the *trans-*Compound **MII**-**OH** [22]. The same space groups, close values of geometric parameters, and similarity in the molecular packing for these three compounds indicate the efficiency of this type of packing methylcyclohexasiloxane molecules. However, an increase in the number of the monomer units with changed conformations in the polymer chain built of such units should be accompanied by changes in their physicochemical properties. In particular, the properties of the resulting polymers can be controlled by choosing the conformational features of monomers taking into account the hydrophilic-hydrophobic nature of the O atoms and CH_2_ groups.

**Scheme 3:**
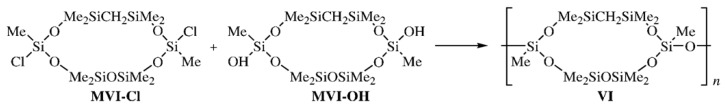
Synthesis of CL PMCS **VI**.

Poly[oxy(2,4,4,6,6,8,10,10,12,12-decamethyl-5-carbacyclohexasiloxane-2,8-diyl)] (**VI**) bearing a CH_2_ unit in the cyclohexasiloxane moiety was synthesized by the heterofunctional polycondensation of Compound **MVI**-**Cl** with the *trans-*isomer of Compound **MVI**-**OH**. The structure of CL PMCS **VI** was confirmed by ^1^H and ^29^Si NMR spectroscopies (Scheme 3) [23]:

Differential scanning calorimetry, X-ray, and optical polarization microscopy studies revealed that CL PMCS **VI** still could form the mesophase and that the ability to self-organize appeared at a polymerization degree of *n* = 14 (Table 2). Earlier, the ability to self-organize with the formation of a 1D mesophase at a polymerization degree of *n* = 3–5 had been reported for CL POS [14]. As the molecular weight of CL POCS **VI** increased to *M*_w_ = 2.55 × 10^4^, *T*_i_ increased to 240 °C (Table 2).

As in the case of CL PMS **II**-**Me**, the X-ray diffraction pattern of CL PMCS **VI** exhibited a strong, narrow reflection at *d*_m_ = 8.4 Å. This suggests a 1D structural organization of the mesomorphic phase in bulk CL PMCS with the Type-**VI** structure.

**Scheme 4:**
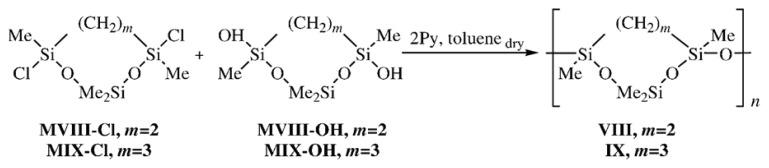
Synthesis of CL PMCS **VIII**, **IX**.

The effect of replacement of an OMe_2_SiO unit rather than single O atom by alkylene (ethylene, trimethylene) groups in the cyclosiloxane moiety of the CL PMCS unit on the ability to form the mesophase through the change in the SiO/SiCH_2_ ratio in the CL PMCS unit was studied taking oligo[oxy(2,2,4,7-tetramethyl-1,3-dioxa-2,4,7-trisilacycloheptane-4,7-diyl)] (**VIII**) and poly[oxy(2,2,4, 8-tetramethyl-1,3-dioxa-2,4,8-trisilacyclooctane-4,8-diyl)] (**IX**) as examples. Cyclolinear PMCS **VIII** and **IX** were obtained by the heterofunctional polycondensation of 4,7-dichloro-2,2,4,7-tetramethyl- 1,3-dioxa-2,4,7-trisilacycloheptane (**MVIII**-**Cl**) and 4,8-dichloro-2,2,4,8-tetramethyl-1,3-dioxa-2,4,8- trisilacyclooctane (**MIX**-**Cl**) with the corresponding dihydroxy derivatives **MVIII**-**OH**, **MIX**-**OH** following Scheme 4 [23]:

The structures of CL PMCS **VIII** (*m* = 2) and **IX** (*m* = 3) were confirmed by ^1^H and ^29^Si NMR data. The ^29^Si NMR spectrum of Polymer **VIII** exhibited two groups of signals, *viz.*, a doublet at δ −15.42 and −15.67 for Me_2_SiO and a triplet at δ −18.99, −19.08, and −19.17 for the MeSi(CH_2_)_2_SiMeO fragment of Polymer **VIII**. The appearance of three signals in a higher field was due to the possibility of three types of joining the units in the polymer chain, namely, *cis*-*trans*, *trans*-*trans*, and *trans*-*cis*. There was no *cis*-*cis* combination of units because heterofunctional polycondensation was conducted using the *trans*-dihydroxy derivative of Compound **MVIII**-**OH**. The ^29^Si NMR spectrum of Polymer **IX**, similarly to that of CL PMCS **VIII**, exhibited two groups of signals shifted to a higher field, namely, a doublet at δ −17.55, and −17.76 for Me_2_SiO, and a triplet at δ −19.91, −19.95, and −19.99 for MeSi(CH_2_)_2_SiMeO.

The properties of Polymers **VIII** and **IX** are summarized in Table 3. DSC and X-ray studies on the physicochemical properties of Polymers **VIII** and **IX** showed that the introduction of alkylene groups into the cyclosiloxane moieties of the CL polymer units lead to the formation of amorphous polymers in the temperature range from −140 to 200 °C despite the fact that Polymers **VIII** and **IX** were enriched with *trans*-*trans* junctions. Polymers **VIII** and **IX** are characterized by the same *T*_g_. The lack of the mesomorphic state in these polymers is most likely due to the asymmetric structure of the silaoxacycloalkane moiety and atactic structure of the polymer chain.

**Scheme 5:**
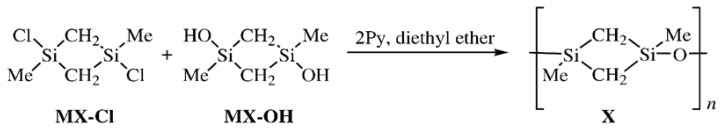
Synthesis of CL PMCS **X**.

**Scheme 6:**
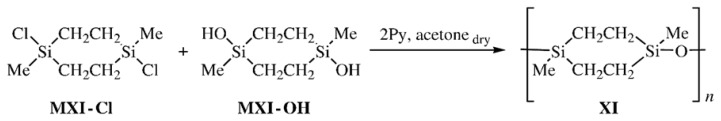
Synthesis of CL PMCS **XI** by heterofunctional condensation.

To compare the physicochemical properties of CL PMCS with asymmetric (**VIII** and **IX**) and symmetric methyldisilaoxacycloalkane moieties, methods of synthesis of bifunctional compounds 1,3-dimethyl-1,3-disilacyclobutane and 1,4-dimethyl-1,4-disilacyclohexane [24,25] were developed. Cyclolinear poly[oxy(1,3-dimethyl-1,3-disilacyclobutane-1,3-diyl)] (**X**) was synthesized by the heterofunctional polycondensation of 1,3-dichloro-1,3-dimethyl-1,3-disilacyclobutane (**MX**-**Cl**) with 1,3-dihydroxy-1,3-dimethyl-1,3-disilacyclobutane (**MX**-**OH**) following Scheme 5 and poly[oxy(1,4-dimethyl-1,4-disilacyclohexane-1,4-diyl)] (**XI**) was obtained in the reaction of 1,4-dichloro-1,4-dimethyl-1,4-disilacyclohexane (**MXI**-**Cl**) with 1,4-dihydroxy-1,4-dimethyl-1, 4-disilacyclohexane (**MXI**-**OH**) following Scheme 6:

Cyclolinear PMCS **X** and **XI** are attractive models for comparing the effects of the ratio of the number of polar to that of nonpolar groups (SiO and SiCH_2_, respectively) and the length of hydrophobic fragments in the unit of CL polymers on the intermolecular interactions in the bulk and in a monolayer on the surface of water.

The crystal structures and molecular geometries of Compounds **MX**-**OH** [21] and **MXI**-**OH** [20] whose conformational features should be retained in the unit of the polymer chain were studied by X-ray analysis.

**Figure 4:**
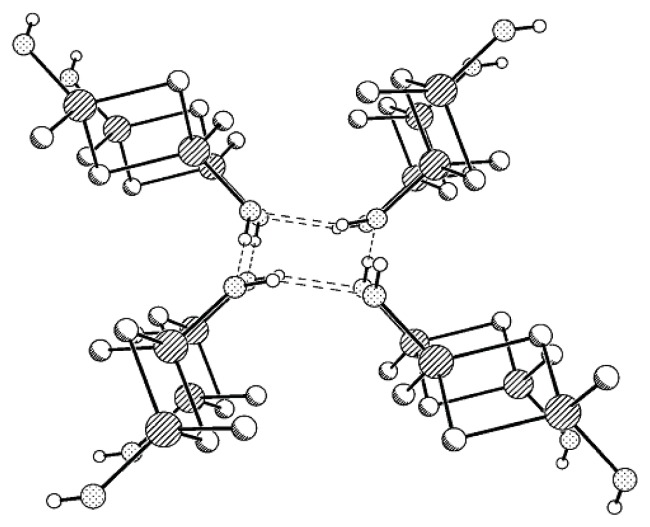
Scheme of chain packing in the crystal of Compound **MX-OH**.

Compound **MX**-**OH** forms H-bonded associates containing two types of independent molecules. Four **MX**-**OH** molecules participate in the formation of a four-membered H-bonded ring, but, unlike Compounds **MII-OH**, **MVI-OH**, and **MVII-OH** characterized by layered packing type, in the structure of **MX-OH** the plane of one disilacyclobutane moiety makes some angle with the plane of the other disilacyclobutane moiety, and this pattern is repeated along the axis (Figure 4).

**Figure 5:**
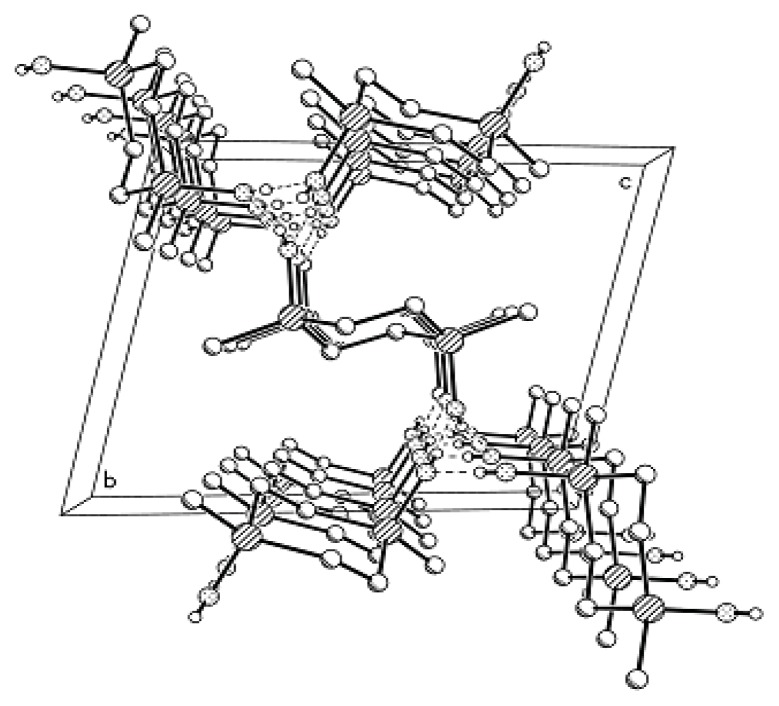
Molecular packing in the crystal of Compound **MXI-OH**. Dashed lines denote intermolecular H-bonds that link molecules to form columns along the *x*-axis.

In the crystal structure of Compound **MXI-OH**, the unit cell contains three independent molecules with very similar conformations to the disilacyclohexane moieties adopting the chair conformation (Figure 5). Silicon atoms deviate from the mean plane of carbon atoms. Here, one deals with the classical tetrahedral valence angles at carbon atoms. In the crystal, the **MXI-OH** molecules form columns linked by an infinite system of H-bonds along the axis. As in Compound **MX-OH**, the centers of three H-bonded **MXI-OH** molecules do not lie in the same plane.

Thus, the columnar crystal structures of the *trans-*dihydroxy derivatives of methyldisilacycloalkanes are fundamentally different from the layered structures of Compounds **MII-OH**, **MVI-OH**, and **MVII-OH**.

X-ray and gel permeation chromatography (GPC) studies showed that the CL PMCS with the Type-**X** unit structure was a completely soluble, amorphous polymer characterized by a polydispersity index *M*_w_/*M*_n_ of 2.57 and *M*_w_ = 1.01 × 10^5^.

**Figure 6:**
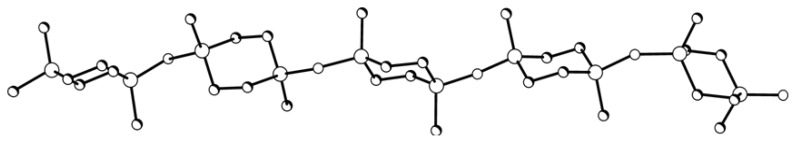
The most probable chain conformation of CL PMCS **XI** determined by the molecular mechanics method.

**Scheme 7:**
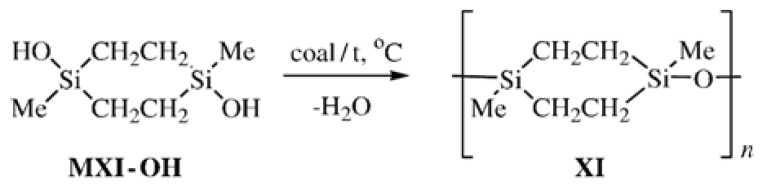
Synthesis of CL PMCS **XI** by homocondensation of compound **MXI-OH**.

Cyclolinear PMCS **XI** was synthesized by the heterofunctional polycondensation following Scheme 6 and by the homocondensation of a mixture of isomers of Compound **MXI-OH** in the presence of activated carbon following Scheme 7:

The ^1^H NMR spectrum of Polymer **XI** exhibits a singlet from CH_3_ protons in the region δ 0.03 or a triplet at δ 0.024, 0.028, 0.030 and a multiplet from CH_2_ protons in the region δ 0.74–1.28. The ^29^Si NMR spectrum of Polymer **XI** demonstrates three multiplets with the same Δδ values (0.05–0.07 ppm) instead of a triplet or quartet with different signal intensity ratios. The multiplet pattern of the ^29^Si NMR spectrum of CL PMCS **XI** reflects the variety of inequivalent silicon atoms in the polymer backbone. Each group of signals corresponds to different combinations of cyclohexane moieties in the polymer chain and characterizes the presence of three conformers with axial, equatorial, and axial-equatorial arrangement of substituents in the chain. The most probable conformation of the polymer chain of CL PMCS **XI** was calculated by the molecular mechanics method. A chain segment of Polymer **XI** comprising five monomer units shows no translational ordering of disilacyclohexane moieties relative to one another along the chain (Figure 6).

Based on the NMR spectroscopy, infrared (IR) spectroscopy, and GPC data, the CL PMCS **XI** with the most regular structure was obtained by the heterofunctional polycondensation (Scheme 6). X-ray data for the molecules of Compound **MXI-OH** in the crystal [20] suggest another type of association compared to that found for Compounds **MII-OH**, **MVI-OH**, and **MVII-OH**. Compound **MXI-OH** goes into the mesomorphic state upon melting [25]. The X-ray diffraction pattern of Compound **MXI-OH** obtained at 132 °C shows no reflections from the crystal and only a narrow reflection at 2θ = 8.86° (*d*_1_ = 9.97 Å) remains. The DSC trace of Compound **MXI-OH** exhibits two endothermic peaks in the temperature range of 124–160 °C. The peaks are shifted toward lower temperatures on re-heating. Unlike Compound **MXI-OH**, the DSC traces of CL PMCS **XI** show *T*_g_ = −54 to −55 °C irrespective of the method of synthesis, and only the syndiotactic Polymer **XI** obtained from pure *trans*-isomers of the monomers demonstrates an endothermic transition with Δ*H* = 1.1 J g^−1^ in the temperature range of 30–58 °C.

### 2.2. The Bulk Behavior of CL POS and CL PMCS

Tables 1, 2 and 3 present the properties of CL Polymers **I**–**XI** with different numbers of SiO and SiCH_2_ groups in the unit. As can be seen that the molecular weights of all CL polymers are not high and that the degree of polymerization varies from 14 to 175. For some CL polymers (e.g., **II** and **VI**), different molecular weights are given, and it is shown that the *T*_i_ values of the polymers increase with increasing the molecular weight. Polymers **II** and **VI** with different numbers of SiO and SiCH_2_ groups are characterized by close *T*_i_ values. The ability of CL PMS **VI** to form the mesomorphic phase appears at much lower degrees of polymerization compared to linear polydiorganosiloxanes, e.g., polydiethylsiloxane and polydi-*n*-propylsiloxane [15,16].

A comparison of the *T*_g_ and *T*_i_ values for the CL POS **I-Me** and **I-Et** and CL POCS **III-Me** and **III-Et** shows that *T*_g_ considerably increases upon replacement of the oxygen atom by the CH_2_CH_2_ unit due to a higher flexibility of the >SiMeOMeSi< group compared to the >SiMeCH_2_CH_2_MeSi< fragment. The CL POCS bearing methyl substituents are prone to crystallization. Unlike CL POS **I-Me**, Polymer **III-Me** does not undergo a transition to the mesomorphic state upon melting. If the introduction of ethyl substituents into the CL POS **I-Et** causes an abrupt extension of the existence region of the mesophase, *T*_g_ of CL POCS **III**-**Et** is reduced by more than 40 °C, but the polymer is in the amorphous state unlike **I-Et**. Thus, the introduction of the CH_2_CH_2_ bridge between the cyclotetrasiloxane moieties causes changes in the mutual arrangement of the two ring planes at a fixed angle ∠ SiCH_2_CH_2_, whereas in the case of CL POS **I-Me**, **I-Et**, and **I-Pr**, the angle ∠ SiOSi can vary between 140° and 180° depending on the size of the substituents; this plays the decisive role in the formation of the 1D or 2D type of packing in CL organosilicon polymers.

**Figure 7:**
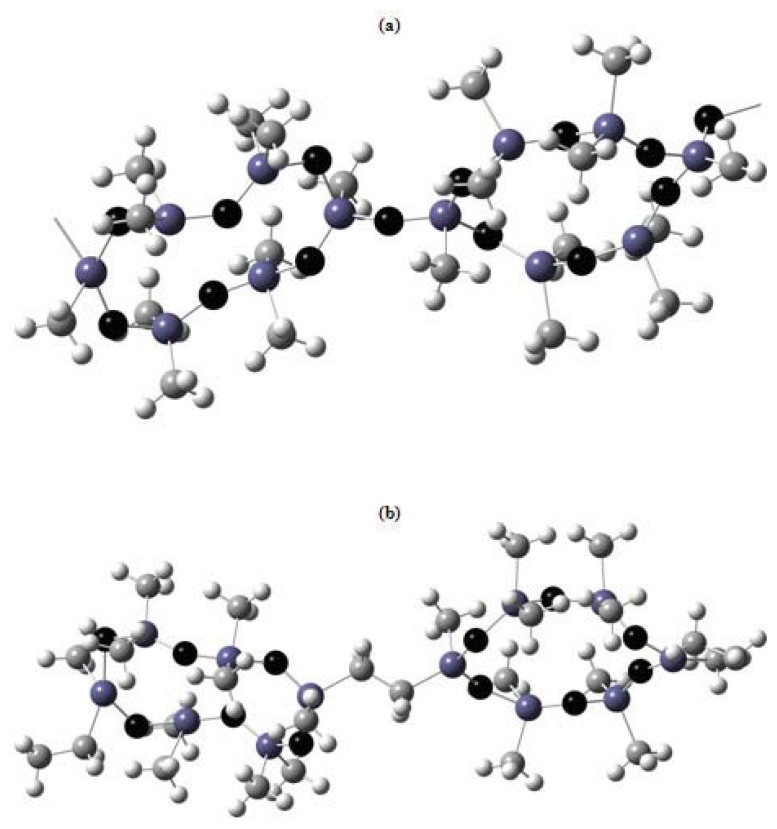
The most probable structure of monomer units in (**a**) CL PMS **II-Me**; and (**b**) CL PMCS **IV-Me** plotted using the Hyper Chem 5 software.

A comparison of the properties of the CL POS **II**-**Me** and **II-MePh** and those of the CL POCS **IV**-**Me**, **IV**-**Et**, and **VI**-**Me** shows that replacement of the oxygen atom in the bridge by a CH_2_CH_2_ unit leads to the loss of the ability to self-organize. At the same time, replacement of one oxygen atom in the cyclohexasiloxane moiety by methylene unit has no effect on the transition temperatures *T*_g_, *T*_i_ and the existence region of the mesomorphic state; therefore, the mutual position of the two moieties in the chain should be considered as the key factor. Note that for Polymer **VI**-**Me** the dependence of *T*_i_ on the molecular weight is the same as that for Polymer **II-Me**. Thus, at the same SiO/SiCH_2_ ratio in the main chain of CL polymethylcarbosiloxanes **IV-Me** and **VI-Me**, the ability to self-organize in bulk is lost for CL PMCS **IV-Me**. However, Compounds **MII-OH** and **MVI-OH** in the crystalline phase adopt a crown conformation and form a layered H-bonded packing. Figure 7 presents the possible types of joining in the CL PMS and CL PMCS backbones. From the data for CL PMS and CL PMCS it follows that replacement of the oxygen bridge between the rings by CH_2_CH_2_ leads to a change in the type of macromolecular packing.

Table 3 lists the properties of CL PMCS **VIII** and **IX** with ethylene and trimethylene groups, respectively, in the trisilaoxacycloalkane moieties. The data in Table 3 show that replacement of the OMe_2_SiO group in CL PMS **I-Me** by aliphatic groups causes *T*_g_ to increase irrespective of the chain length of the (CH_2_)_n_ fragment. However, the *T*_g_ values for Polymers **VIII** and **IX** remain unchanged; above *T*_g_, the polymers are amorphous. A decrease in the number of organic substituents at silicon atom in the methylsilaoxaalkane units leads to weakening of intermolecular interactions. In addition to the Polymers **VIII** and **IX** with asymmetric structures of the chain units, Polymers **X** and **XI** with symmetric structures of the units were obtained. Their properties are also listed in Table 3. Despite the fact that the SiO/SiCH_2_ ratio in the unit considerably decreases (SiO/SiCH_2_ = 0.50 for **X** and 0.25 for **XI**), *T*_g_ changes insignificantly compared to the *T*_g_ values of Polymers **VIII** and **IX.**

Above *T*_g_, CL PMCS **XI** is in the mesomorphic state, as confirmed by the DSC and optical polarization microscopy data. However, *T*_i_ of Polymer **XI** is much lower than those of the CL PMS **I-Me**, **II-Me** or **VI-Me**, although some increase in *T*_i_ of Polymer **XI** with an increase in its molecular weight is quite probable. Nevertheless, the degree of polymerization (*n*) of Polymer **XI** is *n* = 30, being comparable with the *n* values for the two Polymers **VI** (Table 2). A comparison of these data suggests a strong effect of diphilic character of the monomer unit of CL Polymers **I-Me**, **II-Me**, and **VI-Me** on the intermolecular interactions and conservation of 1D ordering in a wide temperature range in these systems.

The aforementioned results are related to self-organization of cyclolinear organosilicon polymers in bulk. Recently, the data on self-organization of *trans*-tactic CL organosiloxane copolymers with different structures of the monomer unit were published [26,27]. In the work [26] the ability of coexistence of two types of macromolecular packing of poly[oxy(2,4,4,6,6,8,10,10,12, 12-decamethylcyclohexasiloxane-2,8-diyl)-dimethylsiloxane] in crystalline and mesomorphic states was demonstrated using different methods. Later, the conditions allowing formation of either one or the other packing were found for the *trans*-tactic poly[oxy(2,4,4,6,6,8,10,10,12, 12-decamethylcyclohexasiloxane-2,8-diyl)-methylvinylsiloxane]. These data will be presented in the next communication.

### 2.3. The Behavior of CL POS and CL PMCS at Air/Water Interface

Earlier [5], it was reported that CL POS could form ordered monolayers. In some cases, destruction of a monolayer is accompanied by the formation of discrete multilayers. The results of the study of the behavior of CL POS on the surface of water show that the chemical structure, namely, the size of the cyclosiloxane moiety, as well as its symmetry and spatial isomerism affects the ability of CL POS to form mono- or multilayers on the surface of water. In addition to these factors, the degree of polymerization and organic substituents at silicon atoms also contribute to the self-organization of CL POS at the air/water interface. A study of the dependence of the surface pressure (π) on the surface area per monomer unit of CL PMS on the size of the cyclosiloxane moiety showed that the collapse surface pressure of the monolayer π was 10 ± 0.5 mN m^−1^ for CL PMS with the chain unit from cyclotetra- to cycloheptasiloxane. It is noteworthy that no unambiguous dependence of π on the number of SiOSi groups in the cyclosiloxane unit of CL PMS was observed [5].

In this work, we studied the influence of changes in the hydrophilic–hydrophobic balance of macromolecules attained by introducing hydrophobic groups into the backbones of two CL POS, namely, poly[oxy(2,4,4,6,8,8-hexamethylcyclotetrasiloxane-2,6-diyl)] (**I-Me**) and poly[oxy(2,4,4,6,6,8,10,10,12,12-decamethylcyclohexasiloxane-2,8-diyl)] (**II-Me**), on:

1. the ability to form a Langmuir monolayer;
2. the limiting surface pressure of monolayer collapse; and
3. the ability to self-organize into multilayer structures upon collapse of the Langmuir monolayer.

Earlier [28], we had studied the effect of hydrophobic groups in 1,3- and 1,4-decamethylcyclohexasilane moieties in CL methylsilanesiloxane copolymers on the surface properties at the air/water interface. It was found that the π value for methylsilanesiloxane copolymers abruptly decreased to 5.0 mN m^−1^ (*cf*. π ≈ 10 mN m^−1^ for CL PMS **I**-**Me** and **II**-**Me**). At the same time, it was shown that 1,3-dihydroxy- and 1,4-dihydroxydecamethylcyclohexasiloxanes could spread at the air/water interface with the formation of monolayers.

**Figure 8:**
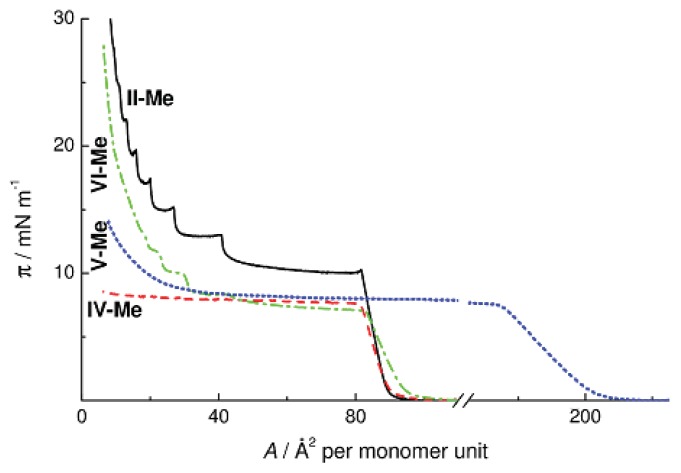
π-*A* isotherms of CL POS **II-Me** and CL PMCS **IV–VI** on water at 20 °C.

Figure 8 presents the π-*A* isotherms of methyl substituted Polymers **II** and **IV**–**VI** with different structures of monomer units. Replacement of an oxygen atom by a hydrocarbon fragment (**IV-Me** and **VI-Me**) causes no changes in the surface area occupied by the monolayer on the surface of water; at the same time, the surface pressure decreases by 2.5–3 mN m^−1^. Thus, macromolecules on the surface are arranged similarly, and the π value decreases because the methylene units are surface inactive. On the other hand, the π-*A* isotherms of Polymers **IV** and **VI** have fundamentally different shapes. Replacement of the oxygen bridge in Polymer **IV-Me** by the hydrophobic ethylene group leads to the loss of the multilayer character of monolayer collapse, whereas replacement of the ring oxygen atom by a methylene unit in Polymer **VI** causes no changes in it. The character of the surface pressure isotherm of Copolymer **V-Me** with ethylene bridges between alternating cyclohexa- and cyclooctasiloxane moieties is similar to the case of CL PMCS **IV-Me**. Thus, it is the inter-ring oxygen atom that determines the character of intermolecular packing of polymer chains in the cyclohexasiloxane monolayers. Replacement of this oxygen atom by the ethylene bridge causes suppression of the mesophase in the bulk of the sample as well as the loss of the multilayer character of the π-*A* isotherm on the water surface for Polymers **IV-Me** and **V-Me**.

**Figure 9:**
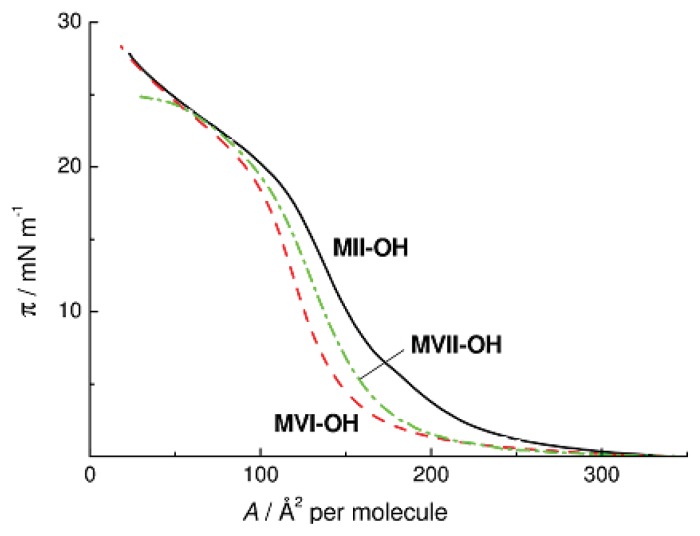
π-*A* isotherms of **MII-OH**, **MVI-OH**, and **MVII-OH** on water at 20 °C.

Our study was in particular aimed at attaining successive replacement of oxygen atoms in the cyclosiloxane moiety by CH_2_ units. Unfortunately, the CL PMCS based on the Monomer **MVII-OH** was not obtained experimentally because an attempt to separate the starting structural isomers **MVII** and **MVII^I^** had failed. In this connection, a comparative assessment was made by studying the behavior of monomeric Compounds **MVI-OH** and **MVII-OH** on the surface of water. Figure 9 presents the π-*A* isotherms of Compounds **MII-OH**, **MVI-OH**, and **MVII-OH**. As can be seen, the three isotherms of the monomeric compounds have similar shapes and close maximum π values. It follows that the major contribution to the interaction with the water surface during association of **MII-OH**, **MVI-OH**, and **MVII-OH** through formation of intermolecular H-bonds comes from OH groups. The SiOSi groups make a less significant contribution to the interaction of cyclosiloxanes with the surface of water.

**Figure 10:**
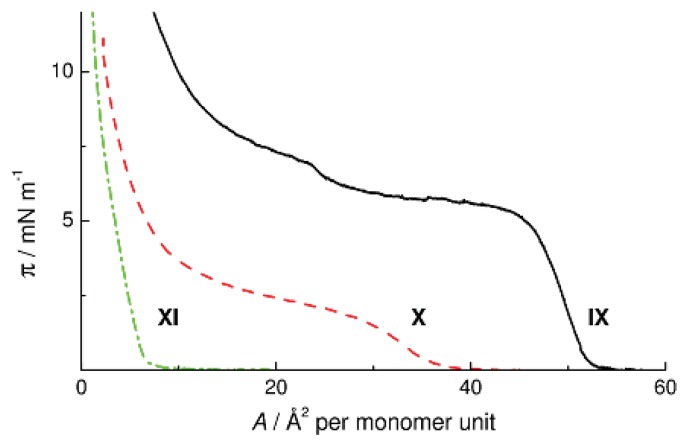
π-*A* isotherms of CL PMCS **IX**–**XI** on water at 20 °C.

Replacement of the OMe_2_SiO fragment of the monomer unit in CL POS **I-Me** by ethylene and trimethylene groups led to Polymers **VIII** and **IX**, respectively. Figure 10a presents a two-step π-*A* isotherm of Polymer **IX** similar to that of the surface pressure isotherm of linear PDMS [8]. By analogy with PDMS, the second transition can be interpreted either as transition to a helical conformation or as the formation of a bilayer, because the pattern of siloxane bonds in the unit of Polymer **IX** is similar to that in linear PDMS and the trimethylene bridge is sufficiently long. The collapse surface pressure of the monolayer of Polymer **IX** decreases to 5.8 mN m^−1^ (*cf*. π = 10.5 mN m^−1^ for CL PMS **I-Me**).

Cyclolinear PMCS **X** and **XI** are attractive models for studying the ability of the polymethylcarbosiloxane chain to form monomolecular layers on the surface of water. In the chains of these polymers, the SiOSi groups are only in the bridges connecting disilacycloalkanes. The π-*A* isotherms of Polymers **X** and **XI** are shown in Figure 10b and c. Polymer **X** forms a stable monolayer which collapses at a surface area of 30 Å^2^ per monomer unit and a surface pressure of 1.8 mN m^−1^, whereas Polymer **XI** does not spread over the surface of water at all. These differences in the surface properties between Polymers **X** and **XI** show that the ability of CL PMCS to form monomolecular films weakens with a decrease in the ratio of the number of hydrophilic to that of hydrophobic groups in the macromolecule. Comparing the behavior of Polymers **X** and **XI**, one should take into account not only the change in the O/CH_2_ ratio, but also the change in the distance between the SiOSi groups in the backbone. By lengthening the hydrophobic segment from 2.63 Å in the 1,3-disilacyclobutane fragment of CL PMCS **X** [24] to 3.44 Å in the 1,4-disilacyclohexane moiety of CL PMCS **XI** [25] we violate the critical ratio that ensures the possibility for the monomolecular film to stay on the surface of water.

**Figure 11:**
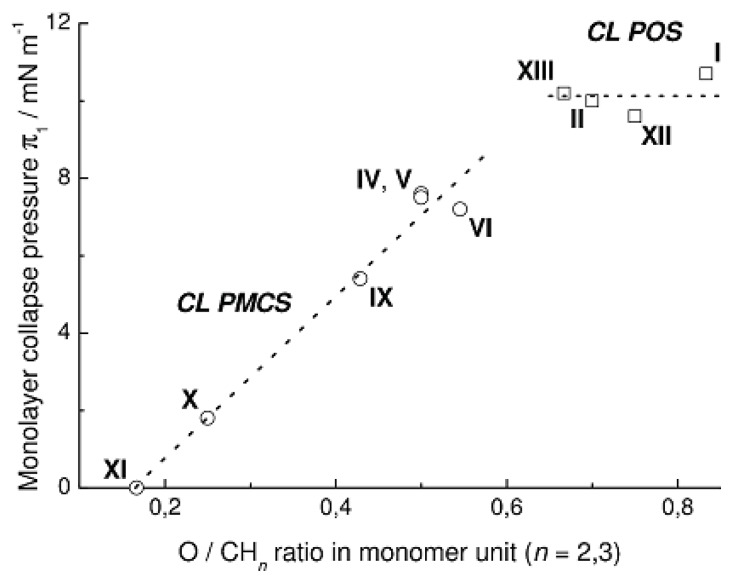
The height of the first step of the surface pressure π_1_ plotted *vs.* ratio of the number of hydrophilic to the number of hydrophobic groups in the monomer unit of methyl substituted CL PMCS. For comparison, the π_1_ values for CL PMS **I-Me**, **II-Me**, poly[oxy(2,4,4,6,8,8,10,10-octamethylcyclopentasiloxane-2,6-diyl)] (**XII-Me**), and poly[oxy(2,4,4,6,6,8,10,10,12,12,14,14-dodecamethylcycloheptasiloxane-2,8-diyl)] (**XIII-Me**) are shown.

Figure 11 presents the dependence of the collapse surface pressure of a Langmuir monolayer on the ratio of the number of hydrophilic (oxygen atoms) to the number of hydrophobic (methyl and methylene groups) moieties in the monomer unit of CL PMCS **IV**–**VI** and **IX**–**XI**. For comparison, we also present the values of the collapse surface pressure of the monolayer π for CL PMS **I**-**Me**, **II**-**Me**, and two other CL PMS with asymmetric central fragment of the chain unit, namely, poly[oxy(2,4,4,6,8,8,10,10-octamethylcyclopentasiloxane-2,6-diyl)] (**XII-Me**) and poly[oxy(2,4,4,6,6,8,10,10,12,12,14,14-dodecamethylcycloheptasiloxane-2,8-diyl)] (**XIII-Me**) [7]. From the data for the series of CL PMS **I-Me**, **II-Me**, **XII-Me**, and **XIII-Me** it follows that variation of the number of siloxane bonds in the polymer backbone (from cyclotetra- to cycloheptasiloxane) does not cause significant variations of the collapse surface pressure of monolayer. At the same time, partial replacement of siloxane bonds in the monomer unit by carbosilane bonds leads to a decrease in the collapse surface pressure of monolayer in proportion to the ratio of the number of hydrophilic to the number of hydrophobic groups in the monomer unit of CL PMCS.

## 3. Experimental Section

### 3.1. Materials

The ^1^H and ^29^Si NMR spectra were recorded on a Bruker P-200 SY spectrometer. The chemical structures of the polymer samples studied in this work are as follows:

Poly[oxy(hexamethylcyclotetrasiloxane-2,6-diyl)] (**I-Me**) was prepared according to published method [1].

The ^29^Si NMR spectrum of Polymer **I-Me** (CCl_4_ + C_6_D_6_ solution) exhibited peaks at δ −19.26, −19.31 (Me_2_SiO); −65.47, −65.53, −65.68, −65.77 (MeSiO_1.5_).

Poly[oxy(hexaethylcyclotetrasiloxane-2,6-diyl)] (**I-Et**) was prepared following a known procedure [4].

The ^29^Si NMR spectrum of Polymer **I-Et** (CCl_4_ + C_6_D_6_ solution) exhibited peaks at δ −19.66, −19.65 (Et_2_SiO); −66.61, −66.65, −66.97, −66.99 (EtSiO_1.5_).

Poly[oxy(hexa-*n*-propylcyclotetrasiloxane-2,6-diyl)] (**I-Pr**) was prepared according to method [4].

The ^29^Si NMR spectrum of Polymer **I-Pr** (CCl_4_ + C_6_D_6_ solution) exhibited peaks at δ −21.96, −21.98 (Pr_2_SiO); −68.13, −68.14 (PrSiO_1.5_).

The intrinsic viscosities and the molecular characteristics of the resulting samples **I-Me**, **I-Et**, and **I-Pr** are listed in Table 1.

Poly[oxy(decamethylcyclohexasiloxane-2,8-diyl)] (**II-Me**) was prepared according to published methods [1,3].

The ^29^Si NMR spectrum of Polymer **II-Me** (CCl_4_ + C_6_D_6_ solution) exhibited peaks at δ −21.95 (Me_2_SiO); −67.55, −67.58, −67.60, −67.63 (MeSiO_1.5_).

Poly[oxy(2,8-diphenyl-4,4,6,6,10,10,12,12-octamethylcyclohexasiloxane-2,8-diyl)] (**II-MePh**) was prepared according to method [2].

The ^29^Si NMR spectrum of Polymer **II-MePh** (CCl_4_ + C_6_D_6_ solution) exhibited peaks at δ −20.70, −21.30 (Me_2_SiO); −80.42, −80.50 (PhSiO_1.5_).

Poly(hexamethylcyclotetrasiloxane-2,6-ethylene) (**III-Me**) and poly(hexaethylcyclotetrasiloxane- 2,6-ethylene) (**III-Et**) were prepared according to method [17].

The ^29^Si NMR spectrum of Polymer **III-Me** (CCl_4_ + C_6_D_6_ solution) exhibited peaks at δ −18.99 (s, Me_2_SiO); −19.13, −9.28, −19.41 [3s, 8:1:1 ratio, MeSi(O)CH_2_CH_2_].

The ^29^Si NMR spectrum of Polymer **III-Et** (CCl_4_ + C_6_D_6_ solution) exhibited peaks at δ −20.22 (s, Et_2_SiO); −20.85 [EtSi(O)(CH_2_CH_2_)_0.5_].

The intrinsic viscosities and the molecular characteristics of the resulting samples **III-Me** and **III-Et** are listed in Table 1.

Poly(decamethylcyclohexasiloxane-2,8-ethylene) (**IV-Me**), poly(decaethylcyclohexasiloxane-2,8- ethylene) (**IV-Et**), and poly[(hexamethylcyclohexasiloxane-2,8-ethylene)-*co*- (tetradecamethylcyclooctasiloxane-2,10-ethylene)] (**V-Me**) were prepared according to a known procedure [17].

The ^1^H NMR spectrum of Polymer **IV-Me** (CCl_4_ + CDCl_3_ solution) exhibited peaks at δ 0.065 (s, 2CH_3_, MeSiO_1.5_); 0.073 (s, 8CH_3_, Me_2_SiO); 0.392 (s, 2CH_2_).

The ^13^C NMR spectrum of Polymer **IV-Me** (C_6_D_6_ solution) exhibited peaks at δ −1.302 (s, CH_3_); 1.189 (s, CH_3_); 8.773 (s, CH_2_).

The ^29^Si NMR spectrum of Polymer **IV-Me** (C_6_D_6_ solution) exhibited peaks at δ −21.59, −21.61 [*trans*, 2Si, MeSi(O)(CH_2_CH_2_)_0.5_]; −21.66, −21.69 [*cis*, 2Si, MeSi(O)(CH_2_CH_2_)_0.5_]; −22.76 [*trans*, 4Si, (Me_2_SiO)]; −22.77 [*cis*, 4Si, (Me_2_SiO)] with a 1.96:1.34 ratio.

No isomeric splitting for Si atoms was observed for Polymer **IV-Me** in a solution in CCl_4_ + CDCl_3_.

The _29_Si NMR spectrum of Polymer **IV-Et** exhibited peaks at δ −22.41 [s, 4Si, (Et_2_SiO)]; −23.09 [s, 2Si, EtSi(O)(CH_2_CH_2_)_0.5_].

The intrinsic viscosities and the molecular characteristics of the resulting samples **IV-Me**, **IV-Et**, and **V-Me** are presented in Table 2.

2,8-Dihydroxy-2,4,4,6,6,8,10,10,12,12-decamethyl-5-carbocyclohexasiloxane (**MVI-OH**): mp 89–91 °C.

The ^1^H NMR spectrum of Monomer **MVI-OH** [(CD_3_)_2_CO solution] exhibited peaks at δ 0.059 (s, 6H, MeSiOH); 0.082, 0.161 (2s, 4H, Me_2_SiO); 0.112, 0.141 (2s, 4H, Me_2_SiCH_2_); 5.33 (OH).

The ^29^Si NMR spectrum of Monomer **MVI-OH** [(CD_3_)_2_CO solution] exhibited peaks at δ 6.12 (Me_2_SiCH_2_); −22.39 (Me_2_SiO); −56.69 [MeSi(OH)O].

2,8-Dihydroxy-2,4,4,6,6,8,10,10,12,12-decamethyl-5,11-dicarbocyclohexasiloxane (**MVII-OH**): mp = 82–83 °C.

The ^1^H NMR spectrum of Monomer **MVII-OH** [(CD_3_)_2_CO solution] exhibited peaks at δ 0.011 (s, 6H, MeSiOH); 0.042 [s, 6H, MeSi(OH)O]; 0.142, 0.160 (2s, 24H, Me_2_SiCH_2_); 5.13 (OH).

The ^29^Si NMR spectrum of Monomer **MVII-OH** [(CD_3_)_2_CO solution] exhibited peaks at δ 5.76 (Me_2_SiCH_2_); −55.73 [MeSi(OH)O].

Poly[oxy(decamethyl-5-carbocyclohexasiloxane-2,8-diyl)] (**VI-Me**), oligo[oxy(2,2,4,7-tetramethyl- 1,3-dioxy-2,4,7-trisilacycloheptane-4,7-diyl)] (**VIII**), and poly[oxy(2,2,4,8-tetramethyl-1,3-dioxy- 2,4,8-trisilacyclooctane-4,8-diyl)] (**IX**) were prepared according to a published procedure [23].

The ^29^Si NMR spectrum of Polymer **VI-Me** (CCl_4_ + C_6_D_6_ solution) exhibited peaks at δ 7.00, 6.97 [(Me_2_Si)_2_CH_2_]; −21.92, −21.94 [(Me_2_Si)_2_O]; −66.42, −66.46, −66.49 (MeSiO_1.5_).

The ^29^Si NMR spectrum of Polymer **VIII** exhibited peaks at δ −15.42, −15.67 (Me_2_SiO); −18.99, −19.08, −19.17 [3s, MeSi(O_0.5_)(O_0.5_)CH_2_CH_2_].

The ^1^H NMR spectrum of Polymer **IX** (Bruker AMX-400 spectrometer, 400.13 MHz, a CCl_4_ + CDCl_3_ solution) exhibited peaks at δ 0.121 [s, 6H, (CH_3_)_2_SiO]; 0.221 [s, 6H, MeSi(O)(CH_2_)_3_]; 0.870 (m, 4H, CH_2_); 1.930 (m, 2H, CH_2_).

The ^29^Si NMR spectrum of Polymer **IX** (CCl_4_ + C_6_D_6_ solution) exhibited peaks at δ −17.55, −17.60 (d, 1Si, Me_2_SiO); −17.75, −17.80 (1Si, Me_2_SiO); −19.91, −19.95, −19.99 [2Si, MeSi(O)(CH_2_)_3_].

The intrinsic viscosities and the molecular characteristics of the resulting samples **VI-Me**, **VIII**, and **X** are presented in Table 3.

1,3-Dihydroxy −1,3-dimethyl-1,3-disilacyclobutane (**MX-OH**): mp 81–82 °C.

The ^1^H NMR spectrum of Monomer **MX-OH** [(CD_3_)_2_CO solution] exhibited peaks at δ 0.210, 0.271 (s, 3H, MeSi); 0.282, 0.349 (2s, CH_2_); 4.780, 4.850 (OH).

The ^29^Si NMR spectrum of Monomer **MX-OH** [(CD_3_)_2_CO solution] exhibited peaks at δ 5.04, 2.23 (2s, 2Si).

*trans*-1,4-Dihydroxy-1,4-dimethyl-1,4-disilacyclohexane (**MXI-OH**): mp 158–160 °C.

The ^1^H NMR spectrum of Monomer **MXI-OH** [(CD_3_)_2_CO solution] exhibited peaks at δ 0.030 (s, 3H, MeSi); 0.740–0.810 (8 m, 8 H, 4 CH_2_); 4.410 (s, 1H, HOSi).

The ^29^Si NMR spectrum of Monomer **MXI-OH** [(CD_3_)_2_CO solution] exhibited peaks at δ 10.57 (s, 2Si).

Poly[oxy(1,3-dimethyl-1,3-disilacyclobutane-1,3-diyl)] (**X**) and poly[oxy(1,4-dimethyl-1, 4-disilacyclohexane-1,4-diyl)] (**XI**) were prepared according to procedures reported in Refs 24 and 25, respectively.

The ^1^H NMR spectrum of Polymer **X** (CCl_4_ + C_6_D_6_ solution) exhibited peaks at δ 0.330 (s, 6H, CH_3_); 0.398 (s, 4H, CH_2_).

The ^29^Si NMR spectrum of Polymer **X** (C_6_D_6_ solution) exhibited peaks at δ 0.25–0.55 (m, MeSi); (−2.37)–(−2.58) (m, MeSi).

The ^1^H NMR spectrum of Polymer **XI** (CCl_4_ + C_6_D_6_ solution) exhibited peaks at δ 0.024, 0.028, 0.030 (t, 3H, CH_3_Si), 0.74–1.28 (m, 8H, CH_2_ cycle).

The ^29^Si NMR spectrum of Polymer **XI** (C_6_D_6_ solution) exhibited peaks at δ 5.32–5.39 (m); 5.42–5.50 (m); 5.58–5.69 (m, CH_2_CH_2_MeSiO_0.5_).

### 3.2. Differential Scanning Calorimetry (DSC)

The phase and relaxation transitions were characterized by a power compensated differential scanning calorimeter Perkin-Elmer DSC-7 according to a conventional procedure. Dry N_2_ gas was purged through the DSC cell. The temperature was calibrated using In an Zn standards, while the heat flow rate was calibrated using in standard.

### 3.3. Wide-Angle X-Ray Diffraction Study

A wide-angle X-ray study was performed with a DRON-3M diffractometer (filtered CuK_α_ radiation, an asymmetric focusing monochromator (a bent quartz crystal)) equipped with a heating and cooling chamber with automatic temperature control within ±1°. The diffraction patterns were recorded in the transmission mode.

### 3.4. Single-Crystal X-Ray Diffraction Study

The crystalline structure of Compounds **MII-OH**, **MVI-OH**, and **MVII-OH** was studied by single-crystal X-ray analysis. The processing of the experimental data and the subsequent calculations were carried out using the SAINT [29] and SHELXTL97 [30] program packages. All structures were solved by the direct method and the non-hydrogen atoms were refined in the full-matrix anisotropic approximation.

### 3.5. Surface Pressure-Surface Area Isotherms

The surface pressure–surface area isotherms were measured by means of the conventional Langmuir trough (FW1, Lauda, Postfach, Germany). The surface pressure was determined by the Langmuir method. Toluene (nanograde, Mallinckrodt Inc., St. Louis, MO, USA) and chloroform (spectroscopic grade, Merck, Whitehouse Station, NJ, USA) were used as solvents. Deionized water purified by Milli-Q system (Millipore Corporation, Billerica, MA, USA) was used as a subphase. The temperature was stabilized with an accuracy of 0.2 °C. The accuracy of the surface pressure determination was 0.1 mN m^−1^.

## 4. Conclusions

Thus, the formation of mesomorphic state in CL PMCS is related to the chemical structure and conformation of the monomer unit. The molecular weight of CL PMCS influences the existence region of the mesomorphic state. On the introduction of an ethylene bridge between the cyclic moieties, the ability to form the mesophase in bulk is lost irrespective of the size of the methylcyclosiloxane unit. However, strengthening of intermolecular interactions due to replacement of methyl substituents by ethyl groups in the case of cyclohexasiloxane leads to recovery of the mesomorphic state with a 2D packing type. The formation of the mesophase in CL PMCS **XI** in a very narrow temperature interval indicates that the contribution of the SiOSi groups in cyclosiloxanes and the ring conformations play the decisive role in the intermolecular interactions in CL PMS **I**, **II** and CL PMCS **VI** in bulk, thus ensuring a thermodynamically stable state in the case of 1D packing of macromolecules.

Unlike the bulk properties of CL PMS and CL PMCS, the behavior of films of these polymers at air/water interface on lateral compression is significantly different. Cyclolinear PMCS **IV**–**VI**, **IX**, and **X** form monomolecular films on the surface of water. However, in most cases, monolayer collapse is not followed by the formation of multilayers similar to the case of CL PMS **I** and **II** [7,8], except for sample **VI-Me**. This correlates with the fact that **VI-Me** is the only CL PMCS studied in this work, which exhibits no disappearance of the mesophase in bulk after replacement of the siloxane group by the carbosilane one. The collapse surface pressure of the monolayer gradually decreases with decrease in the ratio of the number of hydrophilic to the number of hydrophobic groups in the monomer unit of CL PMCS.

## Figures and Tables

**Table 1:** Properties of CL POS **I** and CL POCS **III**, transition temperatures, and packing types.

Polymer	R	[η], dL g^−1^ at 25 °C in toluene	*M*_w_ (*M*_w_/*M*_n_)	*T*_g_, °C	*T*_i_, °C	Packing type in bulk mesophase
**I-Me**	Me a	0.17		−55	105	2D

**I-Et**	Et	0.17		−110	>275	2D
		0.10		−112	180–200	2D

**I-Pr**	Pr	0.23		−50	325	2D

**III-Me**	Me b	0.21	46,000	−30	-	-

**III-Et**	Et	0.08	26,000 (3.1)	−74	-	-

a Syndiotactic chain *T*_m_ = 77 °C;

b *T*_m_ = 42 °C.

**Table 2:** Properties of CL POS **II** and CL POCS **IV**–**VI**, transition temperatures, and packing types.

Polymer	R	[η], dL g^−1^ at 25 °C in toluene	*M*_w_ (*M*_w_/*M*_n_)	*T*_g_, °C	*T*_i_, °C	Packing type in bulk mesophase
**II-Me**	Me	0.10	14,400	−91	200–230	1D

**II-MePh**	Me,Ph	0.17 a	21,900	−50	380–405	1D
		0.49 b			405–420	1D

**IV-Me**	Me	0.13	29,000 (2.08)	−69	–	–

**IV-Et**	Et	0.24	67,300 (3.30)	−104	220	2D

**V-Me**	Me	0.14	34,000	−75	–	–

**VI-Me**	Me	0.15	25,500 (1.62)	−86	210–240	1D
		0.10	11,800 (1.35)	−88	?	
		0.06	6000 (1.38)	−89	68–85	

a CL POS with syndiotactic structure;

b CL POS with atactic structure.

**Table 3:** Properties of CL POCS **VIII**–**XI**, transition temperatures and phase state.

Polymer	[η], dL g^−1^ at 25 °C in toluene	*M*_w_ (*M*_w_/*M*_n_)	*T*_g_, °C	*T*_i_, °C (Δ*H*, J g^−1^)	Packing type in bulk mesophase
VIII	0.06	5000 (1.73)	−60	-	-
IX	0.27	127,000 (8.10)	−60	-	-
X	0.25	101,000 (2.57)	−59	-	-
XI	0.06	4730 (1.52)	−54	30–58 (1.10)	mesomorphic
	0.06	8800 a (8.40)	−56	-	

a CL PMCS **XI** was obtained by the homocondensation reaction.

## Abbreviations

CL: cyclolinear
POS: polyorganosiloxanes
POCS: polyorganocarbosiloxanes
PMS: polymethylsiloxanes
PMCS: polymethylcarbosiloxanes
PDMS: polydimethylsiloxane
